# Food sharing and empathic emotion regulation: an evolutionary perspective

**DOI:** 10.3389/fpsyg.2014.00121

**Published:** 2014-02-25

**Authors:** Thomas R. Alley

**Affiliations:** Department of Psychology, Clemson UniversityClemson, SC, USA

**Keywords:** strong reciprocity, food sharing, empathic emotion regulation, reciprocal altruism, food transfer

The social aspects of food sharing have been largely neglected, particularly in psychology. Mother-infant relations are a notable exception; one where the positive emotional consequences of feeding are rightfully seen as rewarding to mothers. What about food sharing in adults? While much has been written about food sharing as an important form of resource sharing (see Kaplan and Gurven, 2005), scant attention has been given to other consequences although growing evidence reveals other aspects that are more emotional-psychological in nature (Rozin, 1996), including its appearance in courtship (Alley et al., 2013). The potential use of food sharing for empathic emotion regulation (EER) proposed by Hamburg et al. 2014; hereafter HFS) gives us yet another reason to correct this and examine the social aspects of food sharing.

HFS largely ground their proposal in a developmental perspective, suggesting an origin in the positive emotions engendered by food provisioning early in life. Unfortunately it is quite difficult, at best, to connect adult emotional responses to an alleged infantile origin. The EER proposal is supported by various empirical findings, including positive psychopharmacological effects and emotional associations of certain foods as well as emotional eating (Geliebter and Aversa, 2003). Nonetheless, their proposal may prove more powerful if it rested on a more solid foundation. Does adding an evolutionary perspective add credence to their proposal or does it pose a challenge?

An evolutionary perspective on resource sharing leads to the expectation that the provider should expect a favorable cost/benefit ratio in the long run. Something in return is expected that, given its value and likelihood, makes sharing worthwhile (i.e., adaptive rather than maladaptive). The benefits may be obvious in some cases, such as parent-offspring relations, especially if kin selection is considered. Far less obvious cases may occur where the benefits are embedded in reciprocal altruism (Trivers, 1971) with a relatively long time scale such that the benefits may not appear for some time. Moreover, the benefits need not be in kind and may take the form of a vast array of “paybacks” ranging from increases in tolerance to concrete goods or services to improved social status. In addition, the benefits may be potential and not actualized, as in the case of coalition building where the “assets” of others (e.g., in mutual defense) may never be called upon. The point is that there are good reasons to expect food sharing to be done for, broadly speaking, self-interests, even in the case of relatives, mates, and other special individuals. Thus, the reward value of improved emotional state in another may be superfluous and play little or no role in the initiation of food sharing.

Improving the emotional state of another is, however, a benefit that may incur reciprocity. Just as coalitions for defense or for sharing food, for example, can be expected as long as the odds of receiving benefits in return are high enough given the cost of help, so too can positive contributions to affective state. HFS provide a plausible case for expecting positive contributions to affective states of recipients and offer the intriguing suggestion that some reward takes the form of a boost to the provider's affective state, in addition to increasing closeness in the relationship. This view fits nicely with the more general view of emotions as special states shaped by natural selection to increase fitness by enhancing our ability to respond adaptively to opportunities and challenges (Nesse, 1990; Levenson, 1999). A more specific consideration of empathy, however, may lead to the expectation that it serves an adaptive epistemological role as well as a social role. Specifically, empathy may be adaptive due to helpful constraint on predictions of the future actions of others in addition to a serving a social role as a motivator for prosocial behavior (de Vignemont and Singer, 2006). This potential dual function makes it harder to argue for the power of EER to promote food transfer. That is, the primary function of empathy may be to enhance social cognition (i.e., an epistemic purpose) rather than to encourage resource sharing. As such, empathy may promote food sharing by improving recognition of the needs of others even if EER has no direct role in influencing behavior.

Even without reciprocity, EER benefits may motivate food sharing due to a general evolved tendency in our highly social species to help others as long as they too express this tendency. Evidence for this form of prosocial behavior, called “strong reciprocity,” in humans is reviewed by Gintis (2000). With this in mind, the payback provided by EER, even if rather weak in light of ample other reasons for food sharing, may be critical if it is sufficient to push our inherently prosocial tendencies into action. Advocates note that if our species evolved toward strong reciprocity we must have become likely to punish cheaters and non-cooperators as well as to cooperate with others (e.g., Fehr et al., 2002), otherwise cheating and deceit should be widespread and would often render prosocial behavior maladaptive. Such tendencies for punishment should also largely eliminate false emotional expressiveness being used as a device to elicit food transfer.

In conclusion, an evolutionary perspective suggests that caution is needed when interpreting the underlying motivation for food transfers but also provides a solid phylogenetic basis for a limited influence of EER on food sharing.
